# Targeting protein–protein interactions in *Plasmodium*: from asexual replication to sexual development

**DOI:** 10.1186/s13071-026-07390-5

**Published:** 2026-04-18

**Authors:** Jingjing Tang, Wei Tang, Yangxin Xie, Wanyi Yang, Xincheng Luo, Yi Yu, Bo He, Cong Liu, Zhenkui Li

**Affiliations:** 1 Institute of Pathogenic Biology and Key Laboratory of Special Pathogen Prevention and Control of Hunan Province, School of Basic Medical Sciences, Hengyang Medical School, University of South China, Hengyang, 421001 Hunan China; 2 Department of Health Inspection and Quarantine, School of Public Health, Hengyang Medical School, University of South China, Hengyang, 421001 Hunan China

**Keywords:** *Plasmodium falciparum*, Protein–protein interaction, Molecular machinery, Drug discovery, Transmission-blocking, Cryo-electron microscopy

## Abstract

**Background:**

Malaria, a life-threatening protozoan disease caused by Plasmodium parasites and transmitted by mosquitoes, remains a global public health crisis. The 2025 World Malaria Report recorded 282 million malaria cases and 610,000 deaths in 2024, with global elimination goals severely hampered by widespread insecticide resistance and the rapid spread of artemisinin-resistant parasites. Conventional antimalarials primarily target enzyme catalytic sites, which are vulnerable to resistance via single point mutations with minimal parasite fitness cost. In contrast, core Plasmodium biological processes—from erythrocyte invasion and intracellular survival to host-to-vector transmission—are tightly governed by conserved protein-protein interactions. These interfaces have far lower mutational potential, require cooperative compensatory mutations for resistance emergence, and offer high species selectivity, making them promising next-generation drug targets.

**Methods:**

This review systematically synthesizes recent structural and functional advances in key multi-subunit complexes driving the *Plasmodium* life cycle, with a focus on asexual stages of *P. falciparum* and sexual development of *P. berghei* and *P. yoelii*. We integrate insights from cryo-electron microscopy, proximity-dependent biotinylation technologies, and advanced genetic manipulation, and critically evaluate emerging PPI-targeted therapeutic and transmission-blocking intervention strategies.

**Results:**

We delineate the architecture and druggable vulnerabilities of core PPI networks mediating merozoite invasion, intraerythrocytic nutrient uptake, metabolic homeostasis, transcriptional regulation, proteostasis, and merozoite egress in asexual stages. We further dissect PPI networks governing sexual commitment, gametogenesis, fertilization, and mosquito transmission, and summarize the preclinical and clinical development progress of PPI-targeted neutralizing antibodies, vaccine candidates, and small-molecule inhibitors.

**Conclusion:**

Targeting key Plasmodium *PPI* interfaces is a robust, evolutionarily constrained strategy for developing resistance-resilient antimalarials. Technological advances are overcoming the “undruggable” challenges of PPI targets, and this approach holds immense potential to address antimalarial resistance and advance global malaria elimination.

**Graphical Abstract:**

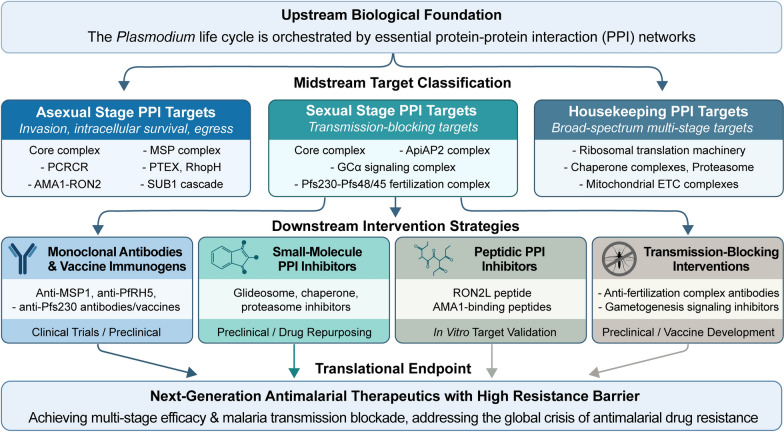

## Background

Malaria persists as one of the world’s most pressing global health crises. Recent data from the World Malaria Report 2025 revealed an estimated 282 million cases in 2024—an increase of about nine million over the previous year—together with a death toll that has reached 610,000 [1]. Even with the ongoing rollout of vaccines such as RTS,S/AS01 and R21/Matrix-M, and despite the wider distribution of insecticide-treated bed nets, moving toward the Global Technical Strategy for Malaria 2016–2030 (reducing global malaria incidence and mortality rates by at least 90% by 2030) remains difficult. This is largely due to the expansion of insecticide-resistant mosquito populations and to the growing resistance of parasites to artemisinin-based treatments [1]. A recent comprehensive analysis of the past, present and future of malaria underscores that while significant milestones have been achieved, the shifting global landscape and the emergence of multi-drug resistance necessitate a paradigm shift toward innovative therapeutic strategies [2].

Most existing antimalarials act on the catalytic sites of specific enzymes. As effective as they may be initially, these strategies are vulnerable because point mutations are common, enabling parasites to escape drug action without incurring a major fitness cost. Chloroquine resistance, for example, is due to mutations in the* Plasmodium falciparum* chloroquine resistance transporter (PfCRT), while artemisinin resistance is usually linked to alterations in the Kelch13 (K13) propeller domain [3]. The other barrier is the parasite’s evolutionary differentiation. This pattern arises because *Plasmodium* species are highly divergent from most commonly used model organisms, and many of their proteins lack homologous domains. In *P. falciparum*, roughly 16% of genes still carry no Gene Ontology annotations, a gap that severely limits the identification of candidate drug targets [4].

The *Plasmodium* life-cycle is highly complex and sophisticated, as it involves transitions between vertebrate hosts (residing in hepatocytes and erythrocytes) and the mosquito vectors [5]. Transitions between different developmental stages are accompanied by distinct morphological transformations in parasites, all tightly coordinated in time and space through extensive protein–protein interactions (PPIs) [6, 7]. Although disrupting PPI interfaces appears to be more challenging than targeting individual proteins, these approaches offer distinct advantages: they tend to be more specific and pose a higher resistance barrier. PPIs are usually based on contacts among multiple non-contiguous amino acid residues, which are cooperative, and their interfaces are not highly conserved across species. This provides a natural structural basis for designing compounds that are directly centered on the parasite while leaving human proteins untouched [8]. In addition, when a crucial interface is broken, it is likely to take co-operative compensatory mutations in both interacting proteins—a phenomenon much rarer than a single point mutation and thus possibly delaying resistance emergence [9].

Driven by advances in technology, the past few years have witnessed a transformative shift in the understanding of the *Plasmodium* protein interactome. Spatial mapping of protein neighborhoods is now possible in living parasites using proximity-dependent biotinylation methods, including biotin identification (BioID), turbo biotin identification (TurboID) and ascorbate peroxidase 2 (APEX2), which reveal local interaction networks that were often omitted in earlier biochemical methods [10–14]. Concurrently, cryo-electron microscopy (Cryo-EM) has elucidated the structures of several large, dynamic complexes in *Plasmodium*, such as the ribosome [15], proteasome [16], apicoplast DNA polymerase [17] and fertilization complex [18]. Together with sophisticated genetic technologies, this progress is gradually leading to the discovery of new key proteins and their functional partners, providing new targets of antimalarial intervention.

In this review, we synthesize current knowledge on protein complexes that drive the *Plasmodium* life-cycle, with particular emphasis on the extensively studied asexual stages in *P. falciparum* and sexual development in the rodent parasites *Plasmodium berghei* and *Plasmodium yoelii*. We examine not only the motor systems responsible for host cell invasion, the metabolic networks required for intracellular growth and the proteostatic machinery essential for survival, but also specialized complexes involved in sexual commitment, gametogenesis and fertilization. Additionally, we evaluate recent advancements to target specific components and/or PPI interfaces of these complexes to develop antimalarial therapeutics. By integrating advances in structural and functional understanding, we aim to develop a well-reasoned framework for the design of transmission-blocking strategies and innovative therapies that are less vulnerable to the development of resistance.

## Key protein complexes in *Plasmodium* asexual stages

### Merozoite invasion

#### Initial adhesion

The initial host–parasite interactions in red blood cells (RBCs) are mainly mediated by members of the merozoite surface protein (MSP) family [19]. In this family, MSP-1,- 2, -4, -8 and -10 are glycosylphosphatidylinositol (GPI)-anchored to the membrane [20], while MSP-3, -6, -7 and -9 could form soluble high-molecular-weight complexes that mediate low-affinity, reversible initial attachment to erythrocytes [21]. The most abundant MSP family member is MSP-1, which is important for initial erythrocyte binding. It is subject to proteolytic processing, producing four fragments of 83, 30, 38 and 42 kDa, respectively [22]. The process of adhesion of MSP-1 to the erythrocyte membrane is mainly based on the interaction between its fragments and certain receptors. The *P. falciparum* (Pf) subtilisin-like protease 2 (PfSUB2) cleaves MSP-1_42_, releasing MSP-1_33_ and MSP-1_19_ [23]. MSP-1_19_ remains anchored to the merozoite surface, facilitating stable adhesion, while MSP-1_42_ and MSP-1_33_ interact with heparin-like molecules on the surface of erythrocytes through their heparin-binding domains [24]. Additionally, MSP-1_83_ binds to the extracellular domain of glycophorin A (GPA) [25], while MSP-1_42_ binds to the non-glycosylated region of the Band 3 protein [26]. It is noteworthy that the Wrb antigen-complex comprises Band 3 and GPA, which are co-receptors and are vital for increasing the efficiency of MSP-1 adhesion through their transmembrane helices [27]. In theory, MSP1 and its interactional partners are promising targets for developing preventive strategies to eradicate malaria. Antibodies to certain elements of the MSP-1/6/7 complex may block the cleavage processing of MSP-1 by PfSUB2 and hinder merozoite invasion [28]. Anti-MSP1 antibodies attach to the p83/p42 subunits of MSP1, which interact with erythrocyte receptors like Band3 and GlyA. These antibodies respond to MSP1’s binding to erythrocytes by creating steric hindrance, preventing merozoites from attaching to RBCs [29]. The corresponding vaccine candidate, SUM101, is currently undergoing clinical evaluation, with phase I trials in malaria-naïve adults having already demonstrated its safety and robust immunogenicity. Recent phase Ib trials completed in Tanzanian adults with prior malaria exposure further confirmed its safety profile and revealed its capacity to boost pre-existing malaria immunity, inducing robust, multifunctional antibody responses. Collectively, these findings position MSP1-targeting antibodies as promising components of next-generation malaria vaccines, although larger-scale efficacy trials in diverse populations are needed to corroborate their protective utility [30]. Interestingly, when combined with optical tweezers-based force spectroscopy, conditional knock-out showed that the loss of PfMSP1 did not significantly change the strength or frequency of merozoite–erythrocyte attachment, suggesting that the protein may not play a major role in initial attachment [31]. Further evidence would be required to reach a clear conclusion on whether PfMSP1 is part of this process. This confirmation might include testing across different strains of *Plasmodium*, improved mutagenesis, blockade of specific protein domains to clarify their roles in attachment and live imaging to monitor localization and interactions. Additionally, comparing the functional redundancy or cooperation of PfMSP1 with other proteins involved in attachment, such as members of the *P. falciparum* erythrocyte binding antigen (PfEBA) and *P. falciparum* reticulocyte-binding homolog (PfRH) families, would provide a clearer understanding of PfMSP1’s specific function during the early invasion stage.

#### Target recognition and membrane deformation

The apical reorientation of the merozoite is a critical step for its invasion of RBCs. This process is precisely controlled by the PhIL1-associated complex (mainly PhIP and GAPM2), which ensures that the merozoite properly reorients itself to establish contact between its apical prominence and the RBC membrane. Such directed contact is a prerequisite for tight junction formation and the initiation of invasion, while also inducing substantial deformation of the erythrocyte membrane [32]. This reorientation is triggered by initial host–parasite interactions mediated by ligands such as the erythrocyte-binding-like (EBL) and PfRH protein families [33]. Key complexes involved in this process include: EBA-175/GPA, EBA-140/GPC, EBL-1/GPB and EBA-181/W from the EBL family, as well as PfRH1/Y, PfRH2a/Z, PfRH2b/Z and PfRH4/CR1 from the PfRh family [19]. Most of these components (such as EBA-175, EBA-140, EBA-181, EBL-1, PfRH1, PfRH2a/b and PfRH4) exhibit functional redundancy, providing alternative pathways for merozoite invasion. EBA-175/GPA, Rh4/CR1 and Rh5/Basigin can enhance RBC deformability and promote invasion by activating the transient receptor potential melastatin 7 (TRPM7) channel on the erythrocyte membrane [34]. In particular, PfRH5 is an essential determinant for erythrocyte invasion; its N-terminal domain interacts with the merozoite surface anchor protein P113, enabling PfRH5 to directly bind the erythrocyte receptor Basigin [35, 36]. This binding causes RBC deformation and releases CyRPA and RIPR from micronemes, which associate with PfRH5’s C-terminus to form the core rolling circle replication (RCR) complex. The RCR complex then assembles with a heterodimer, linked by a disulfide bond between PfPTRAMP and PfCSS, into a larger pentameric PCRCR complex. Following substantial RBC deformation, PCRCR acts as an “inter‑membrane bridge” that stabilizes the expanding contact zone between the merozoite and the erythrocyte membrane, thereby providing an irreversible attachment platform for invasion [37]. After the disassembly of the complex, PfRH5 and RIPR further insert into the erythrocyte membrane, where they may participate in forming transmembrane pores that enable calcium influx. This calcium signal enhances the phosphorylation of erythrocyte cytoskeletal proteins, remodels the cytoskeleton and increases membrane deformability, thereby creating the necessary mechanical conditions for merozoite invasion [38, 39]. In addition, the cleavage of the N-terminal leader domain of PfRH5 by Plasmepsin X activates the invasion function of PCRCR by eliminating steric hindrance at the Basigin-binding site [40]. Notably, the specific interaction between PfRipr and the erythrocyte receptor SEMA7A represents another key mechanism in merozoite invasion [38].

The protein interaction network described above delineates multiple vulnerable sites for therapeutic intervention within the RCR complex, which adopts an elongated conformation. PfRH5 exhibits a distinct kite-like fold, with its apex harboring a defined binding interface for basigin. Although predominantly polar in nature, this interface is functionally essential and highly conserved, rendering it a key site of vulnerability for antibody neutralization; multiple potent neutralizing antibodies, including R5.016 and 2AC7, target this region. PfCyRPA adopts a six-bladed β-propeller architecture and serves as a scaffolding protein that simultaneously engages PfRH5 and PfRipr. Its ligand-binding interfaces are relatively flat and lack characteristic hydrophobic pockets, suggesting that targeting its PPIs with small molecules may be challenging. PfRipr contains multiple EGF-like domains; its C-terminal region (domains 6–8) elicits neutralizing antibodies, although the precise mechanism remains unclear [41].

The structural features of these interfaces dictate the strategies available for therapeutic targeting. Although the PfRH5–basigin interface lacks a defined small-molecule binding pocket, it has nevertheless served as an optimal target for antibody-mediated neutralization, enabling structure-guided vaccine design. Neutralizing antibodies such as R5.004 and R5.016 recognize conformational epitopes at the apex of PfRH5, directly blocking receptor engagement. In contrast, the non-neutralizing enhancing antibody R5.011 binds to a typically disordered flexible region at the N-terminus of PfRH5, prolonging merozoite invasion time by approximately threefold and thereby synergistically potentiating the activity of other functional antibodies [38]. Functional dissection of PfRipr has revealed that antibodies targeting its C-terminal region effectively inhibit merozoite invasion and concurrently block the interaction of PfRipr with both PfRH5 and the erythrocyte receptor SEMA7A. These findings suggest a dual mechanism of action involving disruption of both complex assembly and parasite–erythrocyte recognition [42]. For PfCyRPA, functional studies demonstrate that only antibodies recognizing the native conformation of full-length PfCyRPA can inhibit invasion by disrupting complex formation—specifically by blocking the interaction of CyRPA with either PfRH5 or PfRipr [43]. As expected, the combined administration of anti-PfRH5, anti-CyRPA and anti-Ripr antibodies produces a synergistic inhibition and nearly complete blockade of invasion [44, 45].

Moreover, antibodies that target the RII receptor-binding domain of EBA-175, as well as neuraminidase-mediated enzymatic removal of sialic acids, efficiently block EBA-175 binding to GPA and suppress merozoite invasion. Notably, an antibody targeting this domain has advanced to phase I clinical trials [46–48]. Vaccine-induced antibodies against PfRH2b significantly inhibit invasion by disrupting its interaction with the erythrocyte receptor [49]. Also, small-molecule inhibitors targeting the upstream regulatory protease Plasmepsin X, such as WM4 and WM382, potently inhibit erythrocyte invasion by blocking the precursor processing of PfRH5 and preventing the maturation of the functional PCRCR complex [40].

#### The moving junction

**Fig. 1:**
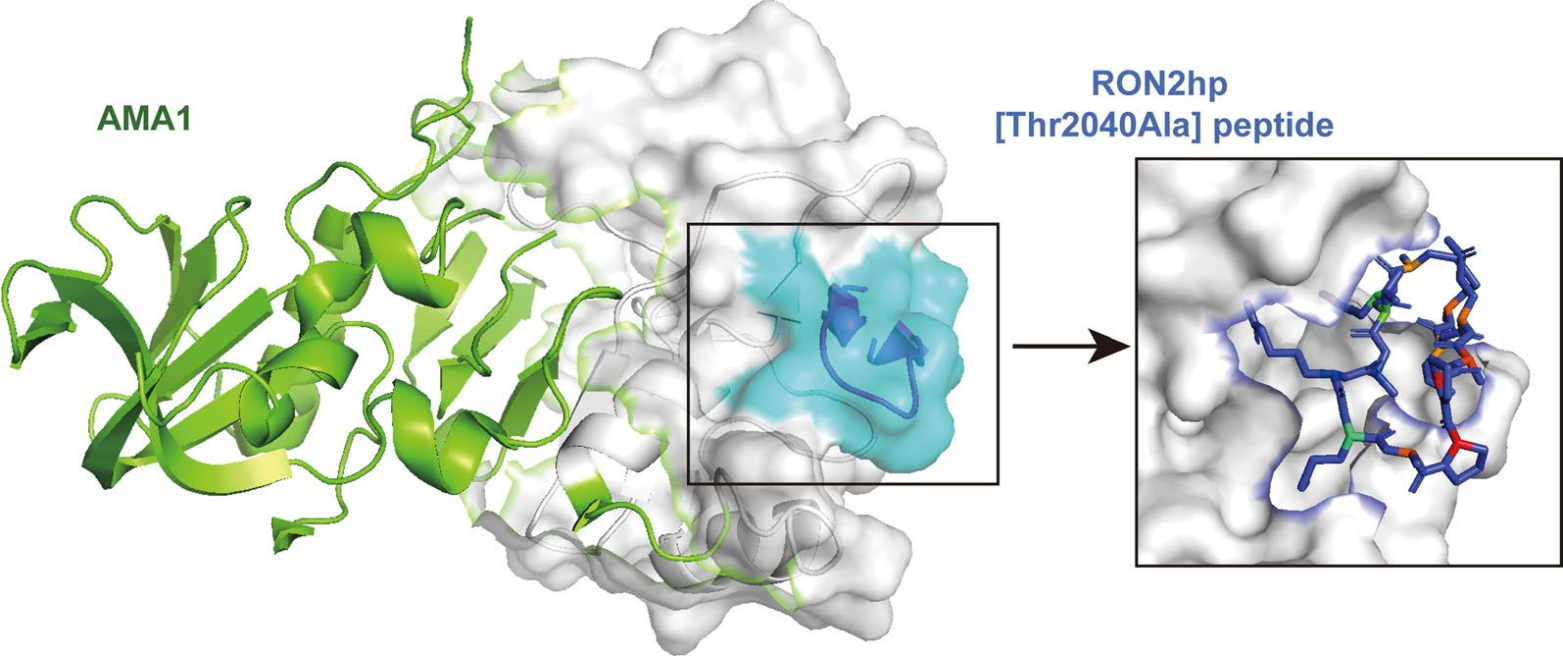
Crystal structure of PfAMA1 in complex with the RON2hp [Thr2040Ala] peptide. The 13-residue β-hairpin based on the C-terminal loop of RON2 (Thr2040Ala) embeds into the hydrophobic groove of AMA1, suggesting the druggable nature of this interface. Peptide DataBank (PDB): 4Z09. PfAMA1, *Plasmodium falciparum* apical membrane antigen 1

The rhoptry neck protein complex (RON complex), composed of RON2, RON4 and RON5, is localized to the rhoptry neck of merozoites and plays a critical role in erythrocyte invasion. Notably, the extracellular loop region of RON2 (RON2L) interacts with apical membrane antigen 1 (AMA1) on the merozoite surface to form the moving junction (MJ) [50, 51]. RON2L, the core binding region of RON2, adopts a stable conformation maintained by a disulfide bond, allowing it to dock specifically into the hydrophobic pocket of AMA1 [52]. Crystal structure analyses reveal that a highly conserved sequence within RON2L adopts a U-shaped conformation, stabilized by a disulfide bond between two conserved cysteine residues, and embeds into the hydrophobic groove formed by domains I and II of AMA1. Key residues, such as Arg2041, engage in specific interactions with conserved amino acids within the groove, thereby ensuring binding stability [51, 53]. Antibodies targeting this hydrophobic pocket (e.g. 4G2 and 1F9), synthetic RON2L peptides and the invasion-inhibitory peptides R1 and F1 can effectively block the AMA1–RON2 interaction and thereby inhibit invasion (Fig. 1). Of note, beyond direct pocket blockade, the recently identified naturally acquired human monoclonal antibody 75B10 achieves broad neutralization of multiple *Plasmodium* strains by targeting a highly conserved epitope outside this pocket, without directly competing with RON2L binding. Furthermore, the structure-based single-component immunogen SBD1, generated by genetically fusing RON2L to AMA1 to precisely lock the complex conformation, does not elicit antibodies that block RON2L binding. Instead, it effectively induces broad neutralizing responses akin to 75B10 and has demonstrated marked strain-transcending protective efficacy in preclinical studies. While most current candidates remain at the stage of target validation and mechanistic exploration, structurally optimized immunogens (e.g. SBD1) and naturally derived human antibodies (e.g. 75B10) have demonstrated promising in vitro activity and broad neutralization, positioning them for further development as vaccine candidates and prophylactic antibodies, respectively [52, 54, 55].

#### The glideosome and actomyosin motor

The glideosome acts as the core machinery that facilitates active invasion in erythrocytes, relying on the precise coordination of multiple components. The glideosome centers around the actomyosin motor module, which consists of class XIV myosin A (MyoA) and two light chains (MyoA tail interacting protein [MTIP] and essential light chain [ELC]) which together form a “dual light chain-regulated” power core. In particular, MTIP supports the structure of MyoA and serves as the main lever arm for transmitting force. Working in concert with MTIP, ELC helps stabilize the neck conformation of MyoA, effectively lengthening the functional reach of the motor’s “lever arm” [56, 57]. This motor module is anchored beneath the plasma membrane via the adaptor protein glideosome-associated protein 45 (GAP45), which binds to the N-terminal domain of glideosome-associated protein 50 (GAP50)—a transmembrane protein embedded in the inner membrane complex (IMC) [58]. Being an IMC-intrinsic protein, GAP50 inserts its transmembrane domain into the outer membrane of the IMC, stabilizing the glideosome [59]. Glideosome-associated protein 40 (GAP40), an IMC-resident protein, associates with GAP50 in the glideosome complex where it stabilizes the expression levels of GAP45 and GAP50, thereby supporting the structural integrity between the IMC and plasma membrane to ensure orderly glideosome assembly [60]. Located on the outer face of the IMC, proteins such as PhIL1, GAPM2, ALV5, the newly identified IMC proteins PIC1–PIC6 and the apical annuli proteins PfAAP2/4/6 assemble into the PhIL1-associated complex, which also exhibits spatial association with the K13 compartment [61]. This complex links to the motor complex (MyoA–MTIP–GAP45) through the C‑terminal region of GAP50, thereby providing structural support to the entire motor system and ensuring its stable anchorage to the inner face of the IMC [32].

During invasion initiation, the merozoite contacts the erythrocyte via surface adhesins like PfRH5 and host receptors, Basigin. To initiate invasion, the parasite secretes transmembrane adhesin MTRAP from its micronemes. The extracellular domain of the adhesin engages host cell surface receptors, while its cytoplasmic tail is physically linked to the parasite’s internal actomyosin system via the glideosome-associated connector (GAC). Serving as a central hub, GAC stabilizes short actin filaments through its N-terminal domain and anchors to the inner leaflet of the plasma membrane by recognizing phosphatidic acid via its C-terminal PH domain. Simultaneously, it binds the cytoplasmic tail of the adhesin, thereby tightly coupling extracellular attachment sites with the intracellular motor machinery [62]. The actin filaments bind to MyoA myosin heads anchored to the IMC. ATP hydrolysis by MyoA drives its directional movement along the actin filaments toward the anterior end of the parasite, generating a rearward force. Since the adhesion protein has already formed fixed anchor points externally, this backward pulling force cannot move the protein itself. Instead, it is converted into a reaction force propelling the entire merozoite forward through the rigid anchoring system GAP45–GAP50–IMC [58]. This demonstrates retrograde movement of adhesion–actin points along the IMC, enabling the merozoite to push forward, breach the erythrocyte membrane and establish contact through the AMA1–RON2 complex to finish entry and form the parasitophorous vacuole (PV) [59]. Such protein complexes represent new therapeutic opportunities for preventing erythrocyte invasion by merozoites. Cytochalasin D directly disrupts actin polymerization, whereas the small molecule KNX-002 binds PfMyoA and inhibits the actin–MyoA interaction, completely halting gliding motility and invasion [63, 64]. Other drugs, including bedaquiline, brilacidin, curine and MMV688271, are GAP50-targeting agents that disrupt the structural and functional integrity of glideosomes and IMC, thereby inhibiting merozoite egress and invasion. Additionally, these agents could also interfere with other critical metabolic pathways through polypharmacological interactions, leading to parasite death [65].

#### The microneme–rhoptry nexus

Malaria parasite invasion of erythrocytes relies on the precise exocytosis of micronemes. This process is driven by the cGMP signaling pathway: environmental signals activate protein kinase G (PKG), leading to intracellular Ca^2+^ release, which in turn activates calcium-dependent protein kinases (CDPKs) to drive microneme membrane fusion [66]. PfCDPK1 mediates the activation of PfPKA, serving as an alternative or supplementary pathway to the cAMP signaling cascade to rapidly initiate the secretion of micronemal proteins (e.g. EBA-175), thereby enhancing parasite attachment and invasion capacity [67]. Although kinase–substrate interactions are not typically classified as conventional PPIs, targeting the kinase active site represents a more prevalent and feasible strategy for pharmacological intervention. Pyrazolopyrimidine compounds such as BKI-1294 and 3-MB-PP1 inhibit CDPK1 activity by competitively binding to its ATP-binding pocket, thereby blocking the calcium-dependent signal transduction required for parasite invasion. These compounds exploit the unique additional hydrophobic space created by the small-volume glycine residue at the gatekeeper site (e.g. Gly128) in CDPK1. The bulky C-3 substituents of these compounds (e.g. the 6-alkoxy-2-naphthyl group in BKI-1294) are accommodated within this pocket, whereas most human kinases possess larger gatekeeper residues (e.g. threonine, methionine) that cannot accommodate such moieties, thereby achieving high selectivity. Among these, BKI-1294 has demonstrated superior pharmacokinetic properties in preclinical studies, including favorable oral bioavailability, metabolic stability, substantial tissue distribution and the ability to achieve therapeutic concentrations in vivo far exceeding its in vitro half maximal inhibitory concentration (IC_50_) values. In contrast, compounds such as 3-MB-PP1 currently serve primarily as chemical biology tools to validate the critical functions of CDPK1 in parasite invasion and intracellular development, with their development still at the in vitro discovery stage [68–70].

**Fig. 2:**
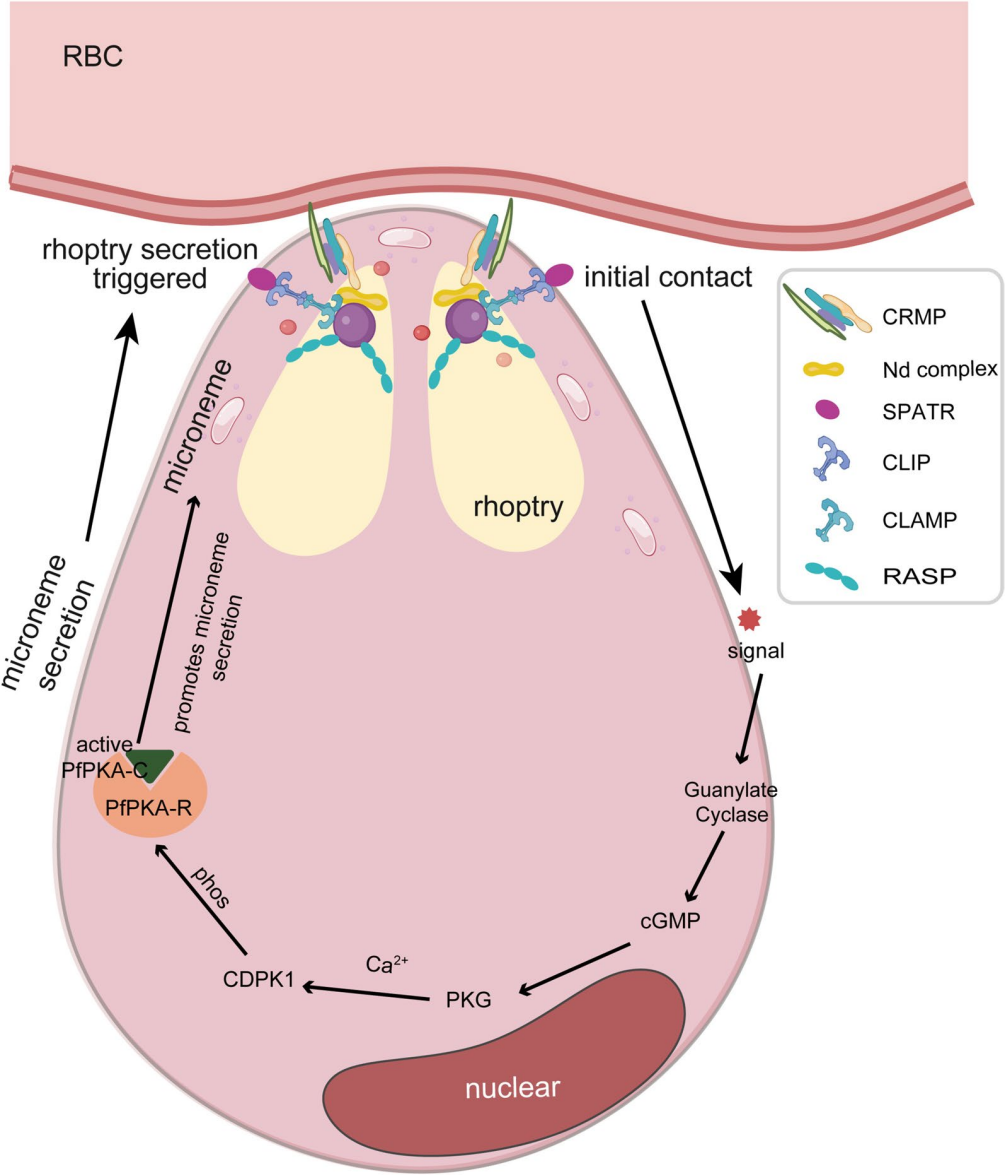
CDPK1 signaling network regulating apical organelle secretion and erythrocyte invasion in *Plasmodium.* Environmental signals activate the central kinase CDPK1 via the cGMP–PKG–Ca^2+^ cascade, which phosphorylates downstream substrate complexes to trigger the release of microneme proteins. Subsequently, the signal is transmitted through conserved multiprotein complexes to initiate the secretion of rhoptry proteins and their exocytosis. This figure was created using BioRender and modified with Adobe Illustrator. RBC, Red blood cell

Rhoptry secretion is triggered after microneme release, and its regulation involves several highly conserved protein complexes and signaling pathways working synergistically [71, 72]. An example is the transmembrane complex of CLAMP, CLIP and SPATR, which serves as one of the main signaling hubs. When SPATR detects host ligands, signals are transmitted through CLIP to CLAMP, ultimately activating downstream rhoptry secretion machinery via its cytoplasmic PRD domain [73]. The CRMP complex is another structural scaffold that detects host ligands through its extracellular domain and transmits signals via its intracellular tail to the highly conserved Nd protein complex. The latter is required to construct the rosette-shaped structure of the rhoptry neck, and its disruption completely disrupts secretion [74–76]. The molecular mechanism governing rhoptry discharge is a pivotal step in host cell entry. Recent evidence highlights the role of the Rhoptry apical surface protein (RASP) complex, which caps the extremity of the rhoptry. Rather than merely acting as a structural component, the RASP complex facilitates the fusion of the rhoptry membrane with the parasite plasma membrane (PPM). This process is potentially driven by the lipid-binding properties of RASP2, which docks the rhoptry to the PPM prior to fusion, thereby enabling the subsequent translocation of the MJ components and other effector proteins [77]. These sequentially coordinated steps constitute a complete regulatory pathway from microneme-mediated signal perception to organized rhoptry release, in which multiple highly specific protein–protein interfaces represent promising new therapeutic targets for intervention against *Plasmodium* invasion (Fig. 2).

### Intraerythrocytic survival

#### Trafficking and nutrient uptake

**Fig. 3:**
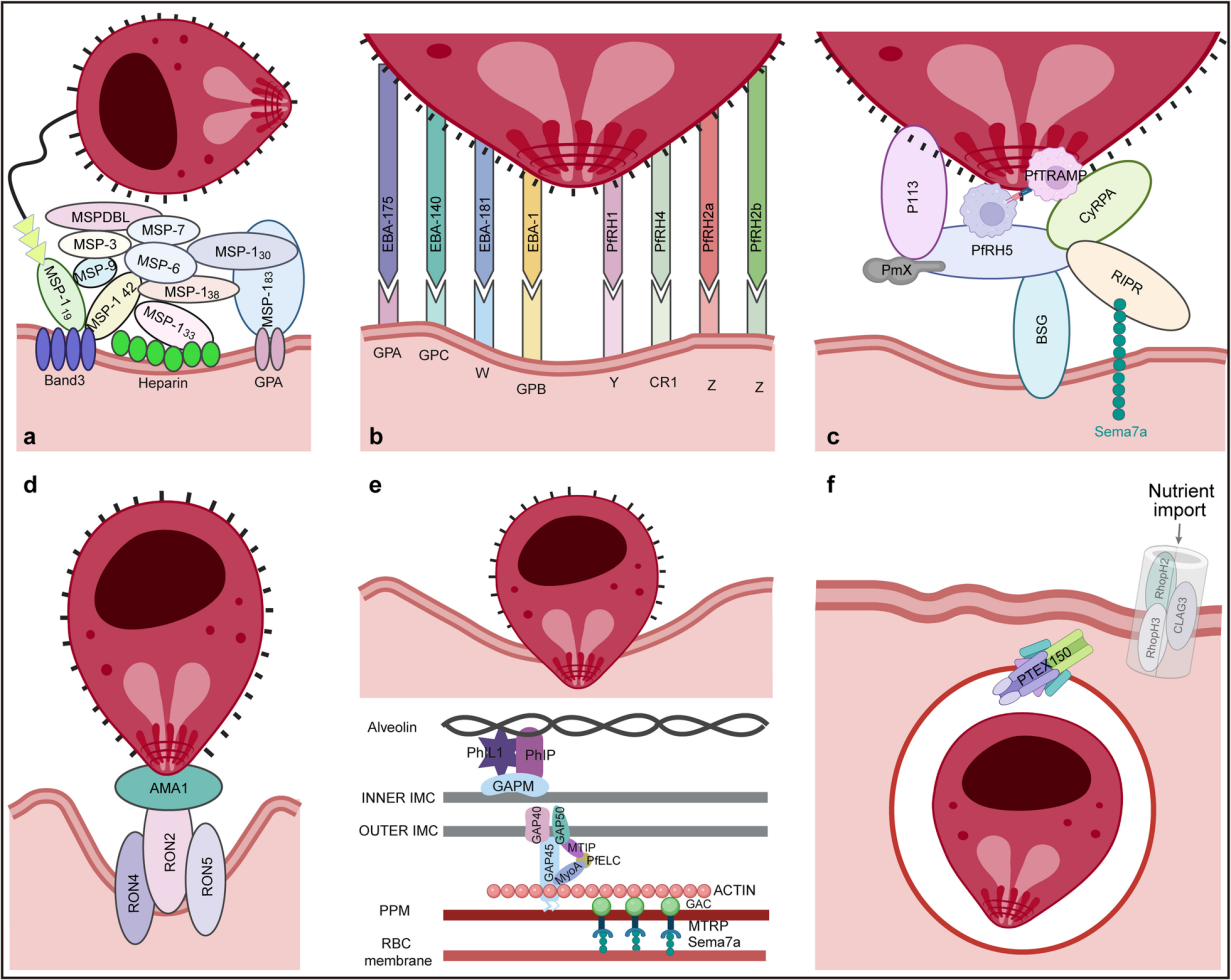
The parasite ligands and erythrocytic receptors involved in merozoite invasion of RBCs. **a** The engagement of MSP ligands with specific receptors on the RBC membrane initiates merozoite adhesion. **b**, **c** Prior to merozoite invasion of erythrocytes, the interaction between its surface ligands and host receptors mediates apical reorientation of the attached merozoite. This process is accompanied by erythrocyte deformation. **d** The RON complex inserts into the erythrocyte membrane, and establishes a tight junction with the MSP AMA1. **e**, **f** Following traversal of the tight junction, the merozoite internalizes, driven by its glideosome complex. This process induces invagination of the host erythrocyte membrane, which envelops the merozoite and forms the nascent parasitophorous vacuole membrane. These figures were created using BioRender and modified with Adobe Illustrator. IMC, inner membrane complex; MSP, merozoite surface protein; PPM, parasite plasma membrane; RBC red blood cell

When merozoites invade erythrocytes, a PV develops around the parasite, surrounded by both the parasitophorous vacuole membrane (PVM) and the PPM [33]. The formation of this structure depends on a crucial rhoptry protein complex—the heterotrimeric Rhoptry H (RhopH) complex, comprised of Cytoadherence-linked asexual gene 3 (CLAG3), RhopH2 and RhopH3 in a 1:1:1 stoichiometry [78]. Structural analyses revealed that the complex adopts a bowl-shaped architecture, stabilized by subunit interfaces, disulfide bonds and shielded transmembrane helices [79, 80]. Biochemical studies suggest that the RhopH complex resides within merozoites as a soluble complex. Following erythrocyte invasion, the RhopH complex—initially delivered to the erythrocyte membrane through rhoptry secretion—is subsequently modified and assembled into the *Plasmodium* surface anion channel (PSAC). This channel facilitates the uptake of nutrients and the release of metabolic wastes, thereby supporting the intracellular survival and replication of the parasite [79–81]. Knocking out RhopH2 or RhopH3 genes causes parasite death and disrupts complex stability, while reducing CLAG3 expression only partially hinders nutrient uptake [82, 83]. This highlights the complex’s essential role in parasite survival, making it a highly promising novel target for antimalarial drug development (Fig. 3).

Following successful erythrocyte invasion, the intracellular survival of the merozoite depends heavily on the *Plasmodium* Translocon of Exported Proteins (PTEX) complex. This essential export machinery, localized to the luminal side of the PVM, comprises HSP101, PTEX150, PTEX88, EXP2 and TRX2 [84–86]. PTEX not only exports parasite effector proteins from the PV lumen across the PVM into the host erythrocyte cytosol, allowing them to reorganize the host cell, but also traffics virulence factors like *P. falciparum* erythrocyte membrane protein 1 (PfEMP1) to the RBC surface where they facilitate endothelial cytoadherence and immune evasion [87]. Changes in its essential interactions, specifically the formation of the EXP2–PTEX150 transmembrane pore subcomplex and the ATPase activity of the HSP101 molecular chaperone, prove to be a significant obstacle to the transport of effector proteins. This export blockade prevents parasite-driven remodeling of the erythrocyte into a growth-permissive niche, ultimately suppressing schizont viability, maturation (including schizont rupture) and the cyclical reinvasion of new erythrocytes by daughter merozoites [88, 89]. Furthermore, disrupting PTEX components, such as HSP101 or EXP2, confirms the significance of PTEX-mediated protein transport systems [85, 90]. These results emphasize that PTEX as a promising target for developing antimalarial drugs. The high-resolution structural analysis of PTEX has provided detailed insights into its molecular structure, serving as an atomic roadmap that guides the rational design of small-molecule inhibitors. These inhibitors can either directly affect the allosteric control of HSP101 or modulate EXP2 pore activity, providing a substantial foundation for the development of a next-generation malaria treatment [89]. Beyond the parasite’s intrinsic PPI-mediated export machinery, the survival of *Plasmodium* is further facilitated by a complex crosstalk at the host–parasite interface. While parasite-encoded complexes orchestrate host cell remodeling, host-derived factors also critically define the immune landscape; for example, it has been demonstrated that host SOD3 suppresses early cellular immune responses to parasite infection [91]. This host-modulated suppression, in conjunction with parasite-driven evasion strategies like PfEMP1 trafficking, constitutes a multifaceted survival mechanism that allows the parasite to thrive within the erythrocytic niche.

To acquire nutrients, the malaria parasite degrades large quantities of hemoglobin, generating cytotoxic free heme, which is then detoxified via a parasite-specific enzymatic system that converts heme into hemozoin. Within the food vacuole, a multiprotein complex of about 200 kDa orchestrates hemoglobin digestion and hemozoin formation. This complex connects crucial hemoglobin-degrading enzymes like falcipain 2/2′ and multiple plasmepsins to the heme detoxification protein (HDP), thereby coupling these two vital processes. Notably, researchers have developed a novel cell-free assay demonstrating that only two recombinant subunits—falcipain 2 and HDP—are sufficient to efficiently convert native hemoglobin into crystalline hemozoin [92]. This system provides new mechanistic insights into the action of critical antimalarial drugs: chloroquine acts as a dual-stage inhibitor, suppressing both hemoglobin degradation and heme polymerization, whereas artemisinin functions as a single-stage agent after heme is released [92]. This work significantly advances our understanding of the parasite’s detoxification pathway and offers a promising new direction for screening novel antimalarial compounds.

The *Plasmodium* K13 protein and its interacting partners—including Eps15, UBP1, AP-2μ and members of the KIC family—localize to the cytostome, where they assemble into a specialized endocytic complex. This complex mediates the uptake of host hemoglobin throughout both the ring and trophozoite stages, thereby providing essential amino acids to support parasite growth. Beyond its role in nutrient acquisition, this endocytic process is critical for the activation of artemisinin and its derivatives (ARTs), as these drugs require hemoglobin degradation products to exert their parasiticidal effect. Resistance-conferring mutations, such as K13C580Y, or conditional inactivation of K13 or its interactors impair endocytic function and reduce hemoglobin uptake. This, in turn, diminishes ART activation and culminates in a resistant phenotype. Although no approved small molecules currently target this protein interaction network, the pathway uncovered in this study presents a promising avenue for antimalarial drug development. Small molecule inhibitors designed to disrupt the interaction between K13 and key effectors such as Eps15 or UBP1 could overcome resistance by both blocking nutrient uptake and restoring artemisinin susceptibility [93].

#### Metabolic complexes

The mitochondrion serves as an essential anabolic center during the trophozoite stage, particularly for the de novo synthesis of pyrimidines. This function is driven by the mitochondrial electron transport chain (mtETC), a network where coenzyme Q/ubiquinone (CoQ) and cytochrome c link upstream dehydrogenases—such as *P. falciparum* NADH dehydrogenase 2 (PfNDH2) and dihydroorotate dehydrogenase (PfDHODH)—to downstream complexes like the cytochrome bc_1_ complex. In these erythrocytic stages, the physiological role of the mtETC is functionally decoupled from oxidative phosphorylation (ATP synthesis); instead, the chain operates primarily as an electron sink to regenerate the CoQ pool required for PfDHODH activity. This specialized dependency on the mtETC for pyrimidine production, rather than ATP generation, establishes a key metabolic vulnerability for parasite growth [94]. Based on the key differences between the nodes of this network and their host mitochondrial counterparts—including the absence of a human homolog for PfNDH2, the distinct conformational features of the PfDHODH binding pocket (where its more spacious hydrophobic channel can accommodate bulky inhibitors, whereas the human ortholog, due to steric constraints, binds only planar molecules) and the local structural specificity of the bc_1_ complex Q site (such as a unique four-residue deletion and a critical hydrogen-bonding network)—a variety of highly selective inhibitors have been developed. These highly selective inhibitors include atovaquone and ELQ-300, which bind the ubiquinol oxidation site (Q_0_) of the bc_1_ complex, preventing electron transfer; the quinolone-based inhibitors CK-2-68 and the optimized analogs SL-2-25 and SL-2-64, which concurrently target both the PfNDH2 → CoQ pool and the CoQ pool → bc_1_ complex, leading to a rapid collapse of mitochondrial membrane potential; triazopyrimidine compounds like DSM1 that selectively occupy the CoQ channel of PfDHODH, disrupting pyrimidine synthesis and mitochondrial function; and DHODH inhibitors such as 5-fluoroorotic acid, which, when combined with atovaquone, improve efficacy and delay resistance. Moreover, acridinedione WR249685 possesses the ability to inhibit both the bc_1_ complex and hemozoin formation, exhibiting greater selectivity than atovaquone. Attacks on key nodes in the ETC interaction network have led to the advance of several candidate molecules, including ELQ-300 and new DHODH inhibitors, into the MMV preclinical pipeline and have provided a solid foundation for the next generation of antimalarials. Among these, atovaquone is a marketed drug, but its physicochemical properties present significant limitations: extremely poor water solubility, low oral bioavailability, the requirement for co-administration with fatty foods to improve absorption, and high plasma protein binding. In contrast, the other small molecules remain at the preclinical candidate stage and require further structural optimization [95, 96].

#### Nuclear architecture and transcriptional control

The parasite’s nuclear architecture and transcriptional machinery rely on intricate protein complexes that regulate gene expression and chromatin organization. Two key systems, nuclear import and chromatin-associated complexes, exemplify how PPIs control gene expression during intraerythrocytic development. Importin α is essential during the asexual stage of *P. falciparum* in RBCs for recognizing nuclear localization signals (NLSs). It heterodimerizes with importin β1, facilitating nuclear import of key proteins like TGS1 and GCN5, regulating gene expression and supporting parasite proliferation. Studies have demonstrated that small molecule inhibitors such as ivermectin and GW5074 can directly bind to *P. falciparum* importin α (PfIMPα), disrupt its α-helical structure and effectively inhibit the interaction of PfIMPα with both NLS and importin β1, thereby blocking nuclear transport and suppressing parasite growth at low micromolar concentrations. Although importin α is functionally highly conserved between parasites and humans, their low sequence similarity provides a potential structural basis for the development of selective inhibitors. Ivermectin demonstrates favorable druggability, with good tissue distribution and a long half-life despite its relatively high molecular weight. As an US Food and Drug Administration (FDA)-approved antiparasitic drug, it shows potential for repurposing as an antimalarial agent. In contrast, GW5074 currently serves only as an in vitro biological tool compound and has not yet advanced to preclinical development [97]. Furthermore, recent studies have uncovered two distinct classes of novel small molecule inhibitors targeting PfIMPα. The first is BAY 11-7085, which exhibits approximately a 12- to 16-fold higher selectivity for PfIMPα over mammalian orthologs; thermal shift assays revealed a unique biphasic effect upon its binding to PfIMPα, suggesting that this selectivity may stem from sequence divergence between parasite and mammalian importin α. The second is the broad-spectrum IMPα inhibitor auranofin, an FDA-approved drug for rheumatoid arthritis, which potently inhibits PfIMPα–NLS interactions at nanomolar concentrations and significantly suppresses the growth of both *P. falciparum* and *Toxoplasma gondii*, underscoring its clinical promise. These findings provide potential therapeutic strategies aimed at disrupting the PfIMPα–NLS interface, with BAY 11-7085 currently serving as a tool compound for proof-of-concept studies, while auranofin presents a clear opportunity for drug repurposing [98].

The Apicomplexan AP2 (ApiAP2) transcription factors have been investigated for their roles in gene regulation and stage-specific development. They form part of two separate complexes. On the one hand, these factors are incorporated into the PfSAGA coactivator complex. PfAP2-LT and PfAP2-I physically associate with PfPHD1 and PfGCN5, respectively, inducing activation of late trophozoite-stage and invasion genes [99–101]. Additionally, stage-specific interactions are clear, as PfAP2-P and PfAP2-EXP/SP physically associate with PfGCN5 only during the ring stage [100]. On the other hand, PfAP2-P, PfAP2-G5 and PfSIP2, along with several uncharacterized factors, are involved in the assembly of the *P. falciparum* microrchidia (PfMORC) complex [102, 103]. This complex localizes to subtelomeric regions and boundaries of chromosome folds, mediating chromatin architecture and gene silencing, particularly at antigenic variant gene families (*var*, *rifin*, *stevor*) [102–104]. Furthermore, the heterochromatin-associated ApiAP2 and PfAP2-HC indirectly bind heterochromatin protein 1 (HP1) and do not exhibit canonical DNA binding; instead, their interactions are related to heterochromatic domain organization [105, 106]. These intricate interaction networks indicate that targeting the interfaces between ApiAP2 transcription factors and their binding partners could effectively disrupt transcriptional regulation and alter chromatin architecture. Such an approach may provide novel therapeutic avenues to impede parasite development and counteract immune evasion.

#### Proteostasis: translation, chaperones and degradation

The mechanism that initiates translation in *P. falciparum* is fundamentally different from that of typical eukaryotes, with its messenger RNA (mRNA) featuring a notably long 5′ untranslated region (UTR) containing numerous upstream open reading frames (uORFs). Instead of hindering translation of the downstream coding sequence, these uORFs could facilitate ribosome re-initiation and flux, thereby supporting highly efficient gene expression [107–109]. This refined mechanism operates through the cooperative regulation of scanning by the translation initiation factor eIF4G1–eIF1 complex and eIF3. The inhibitor i14G1-12 interferes with the eIF4G1–eIF1 interaction, leading to increased leaky scanning, disrupted uORFs spatial organization and blocked re–initiation. This results in ribosomes accumulating at the start site of coding sequences and subsequent global translational suppression, thereby exerting potent antimalarial activity [110]. The quinoline–quinazoline dual-substrate inhibitor, compound 70, directly targets the eIF3i subunit of eIF3. This interaction likely stabilizes the complex in a non-functional conformation, disrupting the precise regulation of translation initiation. As a result, it synergistically suppresses *Plasmodium* protein synthesis and inhibits parasite growth [111].

During translational elongation, eEF1α and eEF2 promote peptide synthesis through their interaction with the ribosome. Specifically, eEF1α delivers aminoacyl-tRNA to the ribosomal A site, supplying the necessary substrates for peptide bond formation. Artemisinin and celastrol can disrupt the interaction between eEF1α and the ribosome, thereby inhibiting nascent protein synthesis in the parasite [112]. Following peptide bond formation, eEF2 is recruited to the large ribosomal subunit in a GTP-bound state and hydrolyzes GTP to provide the energy required for subunit translocation. The (+)-enantiomer of mefloquine directly binds to the GTPase-associated center of the large ribosomal subunit—a hydrophobic pocket formed by ribosomal protein uL13 and the ES13 segment of 28S rRNA—thereby occupying the functional interface of eEF2 and preventing its effective binding and translocation activity. The species selectivity of this inhibition stems from key residue differences in the binding pocket: *P. falciparum* uL13 has a glutamate at position 55 (vs alanine in humans), which forms a critical hydrogen bond with the NH group of mefloquine’s piperidine ring, and a leucine at position 59 (vs lysine in humans), which avoids steric hindrance from the longer lysine side chain in the human ribosome. These two species-specific differences collectively confer a markedly higher affinity of (+)-mefloquine for the *P. falciparum* ribosome compared to its human counterpart, enabling potent parasite killing while effectively avoiding interference with host protein synthesis. DDD107498 (now known as M5717) is proposed to form a stable “poisoning” complex with the eEF2–ribosome–GDP complex, leading to translation arrest and parasite death. As the first antimalarial agent targeting eEF2, this compound demonstrates remarkable selectivity: despite the high conservation of eEF2 across eukaryotes (67.2% sequence identity between humans and *Plasmodium*), DDD107498 exhibits no toxicity to human cells (selectivity index > 20,000-fold). Although the structural basis for this selectivity has not yet been elucidated, the precedent set by the antifungal agent sordarin—which selectively inhibits yeast eEF2—demonstrates that species-specific inhibition of this highly conserved factor is achievable. DDD107498 possesses multi-stage activity (targeting blood, liver and transmission stages), excellent oral bioavailability and a long half-life, conferring potential for single-dose treatment, chemoprevention and transmission blockade. Having advanced to late-stage preclinical development, this compound represents a highly promising antimalarial candidate [113, 114]. Recent in situ cryo-electron microscopy (EM) structural studies confirm that eEF1α and eEF2 interactions with the ribosome occur at specific times and are essential steps in the translation cycle. The investigational drug cabamiquine (CBQ), currently in phase 2 clinical trials, is a *Plasmodium*-specific translation inhibitor distinguished by its multi-stage antimalarial activity, excellent oral bioavailability and favorable safety profile. This candidate selectively inhibits protein synthesis by stabilizing elongation factor eEF2 and disrupting its normal binding cycle with the ribosome. Evidence for its selective targeting of *Plasmodium* is supported by its lack of toxicity to human cells, the loss of function in the eEF2 gene (e.g. the F730L mutation in domain IV) of resistant parasites and thermal stability assays demonstrating that CBQ significantly stabilizes both eEF2 and eEF1α, confirming it as the functional target [115, 116]. Within the endosymbiotic-derived apicoplast organelle, *Plasmodium* uses a prokaryotic-like 70S ribosome translation system. This system remains active throughout the intraerythrocytic asexual stage, particularly in late schizonts, to produce vital proteins necessary for the formation of daughter apicoplasts. The translation complex is made up of prokaryotic-type 30S and 50S subunits. By recognizing mRNAs encoded by the apicoplast genome, it synthesizes enzymes involved in isoprenoid, fatty acid and iron-sulfur cluster metabolism, maintaining the organelle’s metabolic functions and genetic continuity. Low-dose doxycycline inhibits the translation complex of the apicoplast 70S ribosome, specifically targeting the 30S subunit. This action causes a “delayed death” in the second intra-erythrocytic cycle by preventing the translation of proteins required for apicoplast biogenesis in the daughter parasites [117]. *Plasmodium* translation happens in the cytosol and apicoplast using eukaryotic- and prokaryotic-like mechanisms, respectively, both being key antimalarial drug targets. Strategies target initiation/elongation factors, disrupt ribosomal interactions and inhibit prokaryotic translation in the apicoplast, forming a comprehensive approach to block malaria parasite protein synthesis.

In addition to directly inhibiting protein synthesis, exploring and targeting parasite-specific protein folding and homeostasis mechanisms could pave the way for new antimalarial drug development. PfHSP70-1 acts as a central hub in the protein quality control network of *P. falciparum*, orchestrating various chaperones through a dynamic and organized interaction cycle. PfHSP40 family proteins serve as substrate scanners, using their J-domains to recognize unfolded proteins and activate the ATPase activity of PfHSP70-1, thus delivering substrates to its substrate-binding domain for initial folding and stabilization. The nucleotide exchange factor PfHSP70-z catalyzes the exchange of ADP for ATP, driving the conformational resets of PfHSP70-1 and releasing partially folded substrates. Furthermore, the adaptor protein PfHOP bridges PfHSP70-1 and PfHSP90, facilitating the transfer of substrates to PfHSP90 for maturation and activation. This relay-style collaborative network, powered by ATP hydrolysis and exchange, maintains proteostasis under thermal and drug-induced stress, forming a critical molecular basis for parasite survival, proliferation and the development of drug resistance [118]. Despite the high evolutionary conservation of Hsp70, PfHSP70-1 and its co-chaperone HSP40 exhibit key sequence and structural divergences from their human orthologs, establishing a molecular basis for the development of specific inhibitors. At the HSP70 level, PfHSP70-1 possesses unique multi-copy GGMP repeat motifs at its C-terminus. These motifs participate in substrate recognition and interactions with the co-chaperone Hop, and their multi-copy nature constitutes a potential selectivity interface distinct from human homologs. Additionally, PfHSP70-1 features a valine residue preceding its C-terminal EEDV motif, whereas all human orthologs harbor an isoleucine at this position. This single amino acid substitution may subtly alter the conformation of the protein–protein interaction interface, affecting binding specificity with TPR domain-containing proteins. At the HSP40 level, the parasite-specific PfHSP40 exhibits a significantly slower dissociation rate for substrate binding compared to its human ortholog DNAJA1. This kinetic divergence stems from sequence variations in the substrate-binding domains (particularly the C-terminal region), a divergence that enables the parasite chaperone system to more effectively stabilize its aggregation-prone proteome enriched with low-complexity regions. More critically, aspartate 57 within the highly conserved HPD motif of the PfHSP40 J-domain constitutes a key “hotspot” mediating interaction with PfHSP70-1 [119, 120]. These subtle conformational differences and unique sequence motifs enable small molecules to selectively interfere with the parasite chaperone network through differentiated mechanisms without affecting human homologs: the MAL3 series of small molecules directly targets the PfHSP70-1-PfHSP40 interaction interface. 15-Deoxyspergualin binds the conserved EEVD motif, allosterically blocking substrate transfer to PfHSP90 via PfHOP. Additionally, PMB and EGCG inhibit its ATPase activity, thereby dismantling the entire energy-dependent interaction network. Marine prenylated alkaloids (e.g. malonganenones A–C) and naphthoquinones (e.g. lapachol) can competitively bind the substrate-binding domain of PfHSP70-1, blocking its interaction with client proteins and thus impairing its role in maintaining proteostasis [121]. Currently, the aforementioned inhibitors are all in the preclinical discovery phase, primarily serving as molecular probes to validate biological mechanisms. Their pharmacokinetic properties and in vivo safety profiles remain to be systematically optimized and evaluated. Similar targeting strategies have been applied to the PfHsp90 system. As a central hub of the proteostasis network, the function of PfHsp90 is strictly dependent on dynamic interactions with co-chaperones (such as Hop, Aha1 and p23) and its own dimerization to facilitate the folding and activation of client proteins. Currently, small molecule inhibitors targeting this network operate primarily through two mechanisms. The first class comprises ATP-competitive inhibitors, including geldanamycin, harmine derivatives and purine-based compounds (e.g. PU-H71). These agents bind to the N-terminal ATP-binding pocket of PfHsp90, locking it in a closed conformation and indirectly disrupting its functional interactions with co-chaperones and client proteins. The second class directly targets PPI interfaces, including compounds like novobiocin and its derivatives that act on the C-terminal dimerization domain, as well as peptidic molecules that mimic TPR domains. These agents directly block the binding of Hsp90 to co-chaperones or interfere with its dimerization, thereby dismantling the parasite’s protein quality control system. Notably, the latter strategy holds greater potential for selectivity. For example, the convex surface of the TPR2A/2B domains of PfHop forms a critical interface with the middle domain of Hsp90. Sequence analysis reveals that amino acid residues in this region are poorly conserved between *Plasmodium* and humans, providing a structural basis for the development of small molecules or peptidomimetics that specifically disrupt the assembly of the PfHsp90-Hop complex. However, most current small molecule inhibitors targeting PfHsp90 remain in the in vitro discovery phase. Although some compounds demonstrate nanomolar antiplasmodial activity and high selectivity indices, they have not yet advanced to preclinical or clinical trials and are primarily used as molecular tools to validate biological concepts. In contrast, peptidomimetics targeting PPI interfaces face significant pharmacokinetic hurdles. Their large molecular size and poor membrane permeability severely violate Lipinski's rule of five. Coupled with the requirement to traverse multiple biological barriers—including the host erythrocyte membrane, the PVM and the PPM—their potential for clinical translation is extremely low. Currently, they serve only as tools for studying PPIs [122, 123].

TCP-1 ring complex/chaperonin containing TCP-1 (TRiC/CCT) is a eukaryotic class II chaperone that plays a vital role in folding complex proteins such as actin and tubulin. It features a heterohexameric structure comprising eight distinct subunits. Within this complex, the top ring domain—particularly the δ subunit—recognizes and interacts with newly formed or misfolded client proteins, helping them achieve correct three-dimensional structures via ATP hydrolysis. In the asexual stage, TRiC/CCT supports the maturation of cytoskeletal components such as α/β-tubulin, which is essential for the accurate assembly and operation of the mitotic spindle [124]. It has been shown that clemastine binds directly to the crown-ring region of the TRiC δ subunit, thereby competitively inhibiting the specific interaction between TRiC and its substrate proteins. This prevents the effective recognition and folding of tubulin, leading to the aggregation and degradation of unfolded or misfolded proteins. Consequently, tubulin levels in the parasite decrease significantly, and the microtubule structure becomes severely disrupted—ultimately preventing mitosis. Notably, clemastine selectively targets the δ subunit of the *Plasmodium* TRiC/CCT complex. The molecular basis for this species selectivity lies in amino acid sequence differences within the drug-binding region (located in the apical loop domain of the δ subunit) between the parasite and human proteins. These sequence variations result in distinct local conformations of the binding pocket, enabling clemastine to effectively engage and disrupt *Plasmodium* TRiC function while rendering it incapable of effectively binding the human homolog, thereby killing the parasite without significantly affecting human host cells. Although clemastine is an approved antihistamine, its potential as an antimalarial agent remains in the early stage of target validation and in vitro discovery. Similarly, artemisinin has been found to work through a comparable mechanism [125].

The molecular chaperone chaperonin 60 (CPN60) interacts with and stabilizes the caseinolytic protease P (clpP) protease complex in the apicoplast of *Plasmodium*; therefore, it synergistically controls protein folding and ordered degradation to stabilize the apicoplast proteome dynamic equilibrium. Genetic studies reveal that disrupting the CPN60–ClpP interaction triggers rapid disintegration of the apicoplast and induces an “immediate death” phenotype, distinct from the canonical “delayed death” response, thereby underscoring the essential role of this interaction in maintaining organellar integrity. Benzoxazole-based inhibitors targeting the bacterial GroEL ring–ring interface, such as PBZ1587, exhibit low-micromolar to sub-micromolar inhibitory activity against *Plasmodium*. However, definitive evidence confirming its mechanism, involving disruption of a similar interaction interface, has not yet been established [126].

The *Plasmodium* ubiquitin–proteasome system also plays a role in maintaining protein homeostasis during rapid cellular proliferation by targeting misfolded, damaged or functionally obsolete regulatory proteins for degradation. In this pathway, E3 ubiquitin ligases specifically recognize target proteins and tag them with ubiquitin chains. The 19S regulatory particle (or a homologous activator) of the proteasome then recognizes these ubiquitin tags, unfolds the substrate and translocates it into the catalytic chamber of the 20S core particle, where the protein is hydrolyzed [127]. Conventional proteasome inhibitors (e.g. bortezomib, carfilzomib and marizomib) potently inhibit the *P. falciparum* 20S proteasome (Pf20S) but exhibit limited selectivity over the human constitutive proteasome (c20S), rendering them unsuitable as direct antimalarial leads. In contrast, highly selective inhibitors such as J-80 and TDI-8304 achieve potent Pf20S inhibition with markedly reduced activity against the host proteasome. This selectivity is rooted in structural divergences between the Pf20S and c20S β5 subunits: the human β5 subunit adopts a compact, β-sheet-rich conformation with a spatially restricted substrate-binding pocket shaped by a unique “bridged” architecture formed by residues Ala20 and Ala49; in contrast, the *Plasmodium* β5 subunit features an abundance of flexible loop regions, resulting in a more open and plastic binding pocket. These conformational differences are rooted in key amino acid substitutions (e.g. Thr21, Ala22, Tyr169 in humans vs Ser21, Met22, Gly169 in *Plasmodium*), which confer enhanced conformational flexibility and dynamic behavior to the parasite β5 subunit.

 Next-generation inhibitors achieve high species selectivity by precisely targeting these parasite-specific conformational features—for instance, J-80 engages the flexible pocket of Pf20S β5, while TDI-8304 utilizes its macrocyclic scaffold to complement the uniquely open conformation of the parasite proteasome. The AsnEDA class of inhibitors, featuring a non-covalent and reversible asparagine–ethylenediamine scaffold, achieves high selectivity for the Pf20S β5 subunit. Their binding mode involves insertion of the biphenyl moiety into the S1 pocket and its adjacent side pocket of β5, formation of hydrogen bonds between the Asn(*t*-butyl) group and the S3 pocket and engagement of the solvent-exposed S4 pocket (formed by the β6 subunit) via the N-terminal phenylpropionate group. Notably, during in vitro resistance selection with the prototypical AsnEDA inhibitor PKS21004, resistance-conferring mutations did not arise in the direct target, β5. Instead, a single amino acid substitution, A117D, was identified in the adjacent non-catalytic β6 subunit. This A117D mutation is located within an α-helical region of β6, positioned in close proximity to the loop regions that form the S1 side pocket and the S3 pocket wall of the β5 substrate-binding channel. Introduction of the negatively charged aspartate side chain at position 117 creates a steric clash with the neighboring β6 residues Tyr150 and Tyr158, forcing a conformational rearrangement of the local loop. This allosteric remodeling of the β5 binding pocket specifically impairs the binding affinity of AsnEDA inhibitors. Remarkably, this conformational change not only spares inhibitors with distinct binding modes (e.g. bortezomib, carfilzomib) but also markedly enhances sensitivity to the β2-selective inhibitor WLW-VS, a phenomenon termed “collateral sensitivity.” This finding unveils a mechanism of long-range conformational coupling between proteasome subunits mediated by physical interactions and provides the first evidence in *Plasmodium* that targeting a PPI interface can channel resistance evolution through a non-canonical pathway while simultaneously creating synergistic opportunities for combination strategies. This paradigm offers a theoretical foundation for the rational design of resistance-resilient drug regimens. 

Despite these mechanistic advances, however, the pharmaceutical maturity of existing inhibitors as drug-like entities remains limited. Compounds such as J-80, TDI-8304 and the AsnEDA series are generally characterized by high molecular weight and complex peptidic scaffolds, resulting in poor oral bioavailability and substantial violations of Lipinski’s Rule of Five—thereby posing significant obstacles to clinical translation. For example, the representative AsnEDA analog TDI4258 exhibits a short in vivo half-life of approximately 30 min in mice and an oral bioavailability of only 2.3%. To date, only TDI-8304 has demonstrated in vivo efficacy following structure-guided optimization, positioning it as a legitimate lead compound progressing toward drug candidacy [128, 129]. Conceptually more strategic are the artezomibs (ATZs), a class of dual-pharmacophore molecules that precisely intervene across the entire ubiquitin–proteasome axis—from substrate ubiquitination and recognition to proteolytic degradation—thereby converting the parasite’s essential homeostatic machinery into a self-destructive mechanism. However, it must be emphasized that ATZs themselves are not optimized drug candidates but rather tools for validating this innovative biological strategy. Their pharmacokinetic properties—including oral absorption, metabolic stability and safety—require substantial optimization before they can be considered viable antimalarial therapeutics [127].

 In recent years, by integrating multiple computational drug discovery approaches, researchers have identified several FDA-approved compounds, such as Argatroban, Pemetrexed Hydrate, Atazanavir Sulfate and LM-3632, that exhibit high selectivity and strong binding affinity for the *Plasmodium* proteasome. These molecules are promising candidates for novel antimalarial drugs and are particularly relevant to addressing the growing challenge of artemisinin resistance [130]. Notably, artemisinin exerts its antimalarial effect by perturbing proteostasis. Upon activation in vivo, it induces widespread alkylation of parasite proteins, resulting in a surge of aberrant proteins that overloads the proteasomal degradation machinery. When the generation of damaged proteins outpaces their clearance, proteotoxic stress ensues, ultimately leading to parasite death [131]. Similarly, the dynamic, low-affinity interaction between RACK1 and the ribosome serves as a stress-adaptive mechanism. Under conditions such as oxidative stress, this interaction is enhanced, enabling translational reprogramming that prioritizes the synthesis of stress-response proteins. However, artemisinin-induced widespread protein alkylation can hyperactivate this regulatory process—causing RACK1 to become abnormally stabilized on the ribosome. This leads to a collapse of translational programs, blockade of essential protein synthesis and ultimately a complete breakdown of cellular proteostasis [132].

### Schizogony and egress

**Fig. 4:**
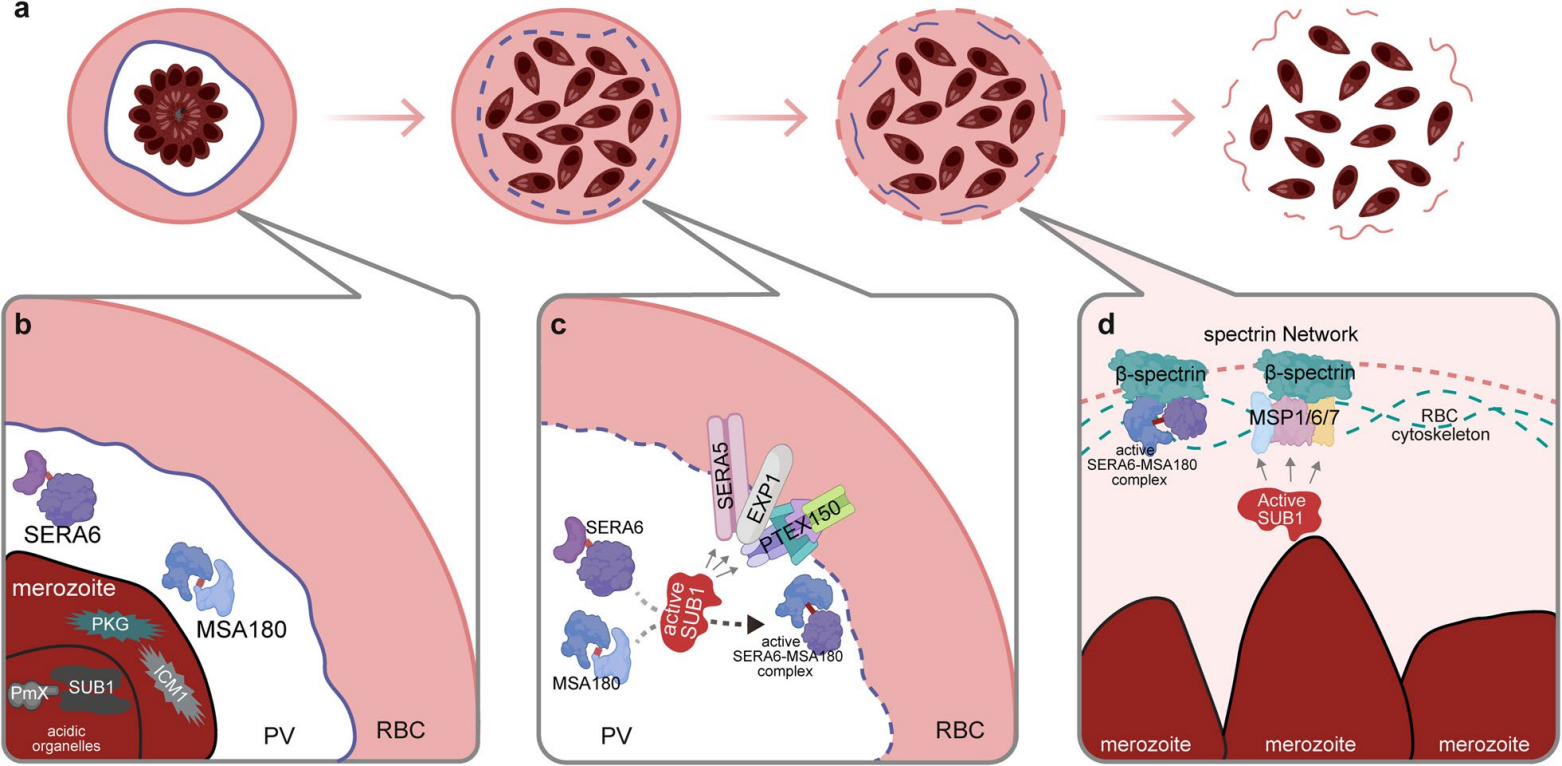
Mechanism of SUB1 protease-mediated proteolytic cascade driving *Plasmodium* merozoite egress. **a** The maturation of schizonts leads to rupture of the PV membrane, followed by disruption of the erythrocyte membrane, ultimately resulting in merozoite egress. **b** In acidic organelles, SUB1 forms a pH-dependent inactive homodimer, but PmX can cleave the inhibitory predomain p31 of SUB1 to directly activate SUB1. **c** Upon entering the neutral PV, SUB1 is activated into a fully catalytically active monomer. The active SUB1 cleaves PV membrane-associated proteins such as EXP1 and PTEX150, as well as SERA5, directly leading to the disruption of the PV membrane. Additionally, it processes SERA6 and MSA180, promoting the formation of the active SERA6–MSA180 complex. **d** Following PV membrane rupture, SUB1 processes the MSP1/6/7 complex, enhancing its binding to the erythrocyte cytoskeletal protein spectrin. The active SERA6–MSA180 complex then synergizes to disrupt the spectrin network, triggering erythrocyte membrane rupture. These figures were created using BioRender and modified with Adobe Illustrator. MSP, Merozoite surface protein; PV, parasitophorous vacuole; RBC, red blood cell; SUB1, subtilisin-like protease 1

Merozoite egress is orchestrated by a cascade of proteolytic events involving multiprotein complexes. Subtilisin-like protease 1 (SUB1), a key serine protease, is maintained as an inactive pH‑dependent homodimer during trafficking but converts to an active monomer upon reaching the neutral PV [66, 133, 134]. Notably, the aspartic protease Plasmepsin X directly activates SUB1 by cleaving its inhibitory prodomain p31 within acidic secretory organelles, revealing a novel activation interface [135]. Once activated, SUB1 cleaves multiple PVM components (e.g. EXP1, PTEX150, SERA5), leading to PVM rupture [136, 137]. Concurrently, SUB1 processes the MSP1/6/7 complex, enhancing its binding to erythrocyte spectrin and anchoring merozoites beneath the inner membrane [138, 139]. Additionally, the serine repeat antigen 6 (SERA6)–merozoite surface antigen 180 (MSA180) complex, generated by SUB1 cleavage, synergistically disrupts the spectrin network to trigger erythrocyte membrane rupture [140]. These proteolytic interactions represent promising drug targets. Small molecule inhibitors such as MRT12113 and the peptidyl boronic acid derivative 3j specifically block SUB1 activity, preventing substrate processing [137, 139]. CWHM‑117 targets Plasmepsin X, disrupting its functional interface with SUB1 and blocking SUB1 activation [135]. Inhibitors like E64d, MMV676881 and triazine nitriles target SERA6 auto‑processing and its interaction with MSA180, hindering β‑spectrin cleavage and egress [140] (Fig. 4). Combining these inhibitors with other egress‑blocking agents (e.g. PKG inhibitors) may enhance efficacy and delay resistance. It should be noted that these small molecule inhibitors currently primarily serve as tool compounds for validating biological mechanisms. As they are still in the early discovery phase, further structural optimization and in vivo validation are required before clinical application.

## Key protein complexes in* Plasmodium* sexual commitment and development

Sexual commitment and development in *Plasmodium* constitute a critical transmission bottleneck for malaria as they are governed by a cascade of molecular interactions. These interactions prove to be essential for accurately carrying out core biological processes—from the initial commitment to sexual differentiation through to zygote formation. In this section, we review key advances in identifying and characterizing the protein complexes that regulate gametocytogenesis, gametogenesis and fertilization.

### Sexual commitment and gametocyte morphogenesis

The epigenetic regulators and the ApiAP2 family of transcription factors work together to control sexual commitment in *Plasmodium*. In the asexual stage of *P. falciparum*, HP1 selectively recognizes genomic locations in which the repressive histone marker H3K9me3 has been deposited. These heterochromatic regions contain families of genes encoding variants of the var antigen, as well as genes encoding secreted proteins that are important to host–parasite interactions [141–143]. Beyond heterochromatin, H3K9me3 and HP1 are found at some euchromatic loci, such as *Pfap2-g* [141, 143]. AP2-G is the main regulator that initiates a transcriptional cascade and turns on early gametocyte genes and sexual differentiation [144, 145]. Additional understanding is based on the observation that the gametocyte development 1 (GDV1) gene interacts directly with HP1 and is generally associated with heterochromatin. Overexpression experiments demonstrated that GDV1 can displace HP1 from multiple genomic sites—especially notable at key early sexual commitment genes like *ap2-g* [146]. AP2-G expression in *Plasmodium* is regulated in a highly complicated manner. In addition to HP1, the repression of *ap2-g* is mediated by a core complex that includes PfAP2-G2 and PfAP2-G5, which interact not only physically but also localize to heterochromatin in a very similar manner. When sexual commitment is initiated, this complex recruits chromatin-modifying proteins such as PfHP1, PfGCN5, and PfMORC to silence the gene of early gametocytes, *Pfap2-g* and others [105, 147, 148]. Functional investigations emphasize the fundamental roles of such factors: PfAP2-G2 knockout halts growth at stage III gametocytes [148, 149], while PfAP2-G5 knockout halts it at stage II despite the higher commitment rates [147]. ApiAP2 members do not function solely as repressors. PfAP2-G4 appears to act as an activator by remodeling chromatin architecture at *Pfap2-g* and decompacting heterochromatin [147]. PfAP2-G interacts with PfAP2-I to help in sexual commitment [150], while PfAP2-P also binds PfAP2-I, indicating that distinct protein complexes are specifically involved in the regulation of host cell invasion and gametocytogenesis [151]. The conserved nature of ApiAP2-mediated transcriptional regulation is further exemplified in the closely related apicomplexan *T. gondii*. While *Plasmodium* utilizes a cascade of AP2 factors to orchestrate sexual commitment, recent evidence suggests that *T. gondii* employs a similar logic to govern life-cycle checkpoints. Specifically, the transcription factors AP2XI-2 and AP2XII-1 have been identified as key negative regulators that suppress merozoite-primed pre-sexual commitment during asexual development [152]. This suppressive mechanism parallels the roles of repressor ApiAP2s in *Plasmodium* (e.g. PfAP2-G2), where transcriptional repression of alternative developmental programs is essential for maintaining stage-specific homeostasis. Such parallels across the Apicomplexa underscore the notion that reliance on multi-protein ApiAP2 complexes to control developmental transitions is an evolutionarily conserved strategy, thereby validating these transcriptional networks as promising targets for broad-spectrum anti-parasitic drug discovery. 

However, although ApiAP2 transcription factors represent attractive drug targets due to their essential role in asexual and sexual development and their absence in humans [153], it is important to acknowledge the considerable challenges they pose for conventional drug discovery. The AP2 DNA-binding domain comprises a three-stranded antiparallel β-sheet stabilized by an α-helix, in which the β-strands mediate sequence-specific DNA recognition through direct contacts with the major groove. This structural architecture appears to lack well-defined ligand-binding pockets. To navigate this “undruggable” landscape, alternative therapeutic modalities may be required. Proteolysis-targeting chimeras (PROTACs) offer a promising strategy: by recruiting an E3 ubiquitin ligase to the target protein, PROTACs could induce the degradation of ApiAP2 factors even in the absence of direct functional inhibition [154]. Furthermore, the identification of small molecules capable of disrupting AP2-EXP DNA-binding activity through virtual screening suggests that the AP2 domain may harbor transient or allosteric pockets induced by DNA interaction or protein–protein assembly, offering a structural rationale for future rational drug design [155].

 Enzymes that modulate epigenetic states have emerged as key therapeutic candidates. Histone acetylation and methylation are dynamically linked. In the example of sexual commitment in *Plasmodium*, the transition from the repressive H3K9me3 mark to the activating H3K9ac mark requires a combination of the actions of the methyltransferase PfSET3 and the acetyltransferase PfGCN5 [156, 157]. This co-operation helps dissociate the H3K9–HP1 complex, thereby derepressing *Pfap2-g* and triggering gametocytogenesis [158]. Conversely, the deacetylation of this gene requires the deacetylase PfHDA2, which removes acetyl groups from H3K9, thereby restoring methylation. These precisely coordinated interactions, crucial for controlling sexual commitment, emphasize the potential of targeting epigenetic regulators with drugs. Therefore, developing “epidrugs” could be beneficial in efforts to eliminate malaria [159].

The IMC serves as a cytoskeletal scaffold that directs morphological changes across both asexual and transmission stages of the parasite [160]. Critical to gametocyte development [161, 162], IMC components are expressed from stage II through stage V [163]. At first, the structure looks like a single, spine-like formation that resembles a “hat” during stage III [164]. As development progresses, it gradually expands to surround the entire cell in stages IV and V, helping to restore the cell’s symmetry [165, 166]. According to recent research, loss of PfBLEB during gametocytogenesis disrupts IMC expansion, microtubule assembly, gametocyte morphology and associated structures. Further discussion reveals that cytoskeletal remodeling components change dynamically during development. Notably, PfBLEB does not interact with proteins typical of the asexual-stage basal complex in gametocyte formation. Instead, it binds to several proteins shared by schizonts and gametocytes, including established basal complex components and uncharacterized proteins, suggesting the involvement of a novel subcellular compartment in this process [167]. PfFBXO1 is also vital for preserving IMC structure and maturing gametocytes. The interaction of PfFBXO1 with PfSKP1 and various IMC-associated proteins has been validated in proteomic studies, with additional evidence that these interactions are part of significant regulatory complexes involved in gametocyte development [168]. However, the proposed PfFBXO1 interactome lacks cullin proteins, unlike the typical SKP1–cullin–F-box (SCF) E3 ubiquitin ligase complex observed in *P. berghei* [169].

### Gametogenesis, fertilization and the transmission interface

Environmental stimuli that cause gametogenesis in *Plasmodium* are a slight drop in temperature, elevated pH and the presence of xanthurenic acid (XA) [170, 171]. Gametogenesis essential protein 1 (GEP1) is crucial for XA-induced gametogenesis in rodent malaria parasites. This protein interacts directly with guanylyl cyclase α (GCα), affecting the enzyme’s subcellular localization, especially in XA-activated male gametocytes [172]. Even when treated with the phosphodiesterase inhibitor zaprinast, GEP1-knockout parasites remain developmentally arrested, confirming that GEP1, which is conserved in both *P. yoelii* and *P. falciparum*, is required for GCα catalytic function [172, 173]. Also, the signaling linking factor (SLF) and the unique GC organizer (UGO) regulate GCα activity, with the deletion of either leading to gametogenesis defects. These proteins create a membrane-associated signaling platform responsive to environmental signals that activates gametocytes. While GEP1 and SLF maintain stable cGMP synthesis through GCα in gametocytes, UGO is particularly essential for increasing GCα production in response to natural stimuli [174]. It has recently been revealed that GEP1 binds XA, and because of its high affinity for this molecule, it is regarded as the primary recognition factor [175]. The essential role of GCα extends to the asexual blood stages of *P. falciparum*, and its loss leads to failure in cGMP synthesis, impaired intracellular calcium mobilization and ultimately an inability of merozoites to egress from host erythrocytes [176]. Given the essential function of the membrane-associated signaling platform involving GEP1 and its partners, such as SLF and UGO, in controlling GCα activity, there is considerable potential to develop antimalarial approaches targeting these proteins or their PPIs.

Sex-specific gene expression in female gametocytes relies on two ApiAP2 factors with female-enriched expression, PyAP2-O3 and PbFD3, although their mechanisms differ substantially [177, 178]. Intriguingly, PbAP2-FG2 (the PyAP2-O3 homolog) and PbAP2R-2 (formerly PbFD3) physically interact, forming a vital transcriptional repressor complex. ChIP-seq analysis revealed widespread genomic co-localization of these two factors, and rapid immunoprecipitation mass spectrometry of endogenous proteins further validated the presence of this complex [179]. The two factors have a conserved C-terminal AP2-coincident domain (ACDC), a four-helix bundle present only in apicomplexans that probably mediates interactions between ApiAP2 proteins (based on unpublished results in our laboratory). An in silico docking screen of established antimalarial compounds revealed five leading molecules that bind to one of the conserved hydrophobic pockets within the ACDC domain [180]. These findings emphasize the promise of ACDC as a parasite-specific drug target and provide a structural rationale for developing broad-spectrum antiparasitic agents.

Translational repression, governed by diverse RNA-binding proteins, represents a crucial post-transcriptional regulatory layer during *Plasmodium* sexual development [181]. Early support for a repressive complex emerged from affinity purification of development of zygote inhibited (DOZI)::GFP in *P. berghei*, which identified CARP interactor with THY domains (CITH) as a partner. Reciprocal co-immunoprecipitation using CITH::GFP identified a 16-component complex containing DOZI, CITH and several ALBA family members, which was named the DOZI/CITH/ALBA (DCA) complex [182]. Subsequent TurboID-based proximity labeling proteomics revealed stage-specific spatial and compositional profiles: while 14 proteins in female gametocytes and 13 in zygotes stably interacted with ALBA4 and DOZI, respectively, only 62% of their associated proteins overlapped in gametocytes, indicating a condensed messenger ribonucleoprotein architecture during the repression stage. Reciprocal DOZI–ALBA4 labeling, in contrast, was barely observed in zygotes, implying that DCA complex disassembly or significant restructuring occurred during translational release. Of the mRNAs that have been translated, 198 transcripts are repressed in gametocytes, subsequently becoming actively translated in zygotes. These transcripts encode mitochondrial respiratory chain components, the IMC proteins and the transcriptional regulator ApiAP2-Z, all of which are essential for survival at the mosquito stage. Taken together, the storage of these mRNAs coupled with the dynamic remodeling of the DCA complex constitutes a regulatory network that guides parasite development [183]. Such findings illustrate how *Plasmodium* finely tunes gene expression by dynamically reconfiguring protein complexes across life stages—a clear example of the “dynamic network” paradigm. Therefore, targeting this spatio-temporal specific dynamic translation repression complex is expected to lead to the development of new antimalarial drugs by disrupting the regulatory network of gene expression essential for host-vector transmission.

Osmiophilic bodies (OBs), which are membrane-bound organelles in gametocytes, serve as primary reservoirs for secretory proteins essential for egress during gametogenesis [184]. Multiple OB-resident factors—including G377 [185], MDV1/PEG3 [186, 187], GEST [188] and GEP [189]—have been linked to sexual development. Kehrer et al. identified a distinct proteome of secreted proteins using a vesicular BioID technique by tagging OB proteins with MDV1/PEG3 in vivo [10]. Based on this finding, Sassmannshausen et al. compared proteomes of OBs and PPLP2-negative egress vesicles (P-EVs) in *P. falciparum*. Their results showed that OB exocytosis is calcium-independent and precedes P-EV discharge, whereas P-EV exocytosis is calcium-dependent, suggesting different regulating mechanisms. Moreover, the analysis of BioID with the bait proteins G377, MDV1 and PPLP2 identified 143 gametocyte egress vesicle proteins, 20 of which were shared by all three baits. STRING network analysis highlighted important protein clusters, including an adhesion complex containing LCCL-domain proteins and a vesicular trafficking cluster containing the Vti1-v-SNARE Vti1, indicating the presence of large interaction networks [190]. Through these studies, researchers have defined OBs as crucial participants in parasite egress and have identified mechanisms in *Plasmodium* species that may inform the development of transmission-blocking strategies.

Pfs230 and Pfs48/45 are both found in gametes and gametocytes. The interruption of either gene has a drastic effect on oocyst formation, underscoring the latter’s role in determining mosquito infection [191, 192]. These proteins were first discovered as targets of antibodies with potent transmission-reducing activity [193, 194]; however, they have entered a new paradigm bridging interaction to intervention. The cryo-EM structure of the Pfs230–Pfs48/45 fertilization complex has been determined, providing the first detailed insight into this key interaction: the C-terminal domains 13 and 14 of Pfs230 (D13D14) serve as the exclusive binding sites for all three domains of Pfs48/45. This is a non-redundant interaction, as the deletion of D13D14 blocks Pfs230 surface localization and almost completely inhibits oocyst formation. Mechanistically, nanobodies with specificity for the D13D14 interface, such as W2809, which sterically prevent the Pfs48/45 D1Pfs230 D14 interaction, inhibit the complex in vitro. These structural insights have encouraged the development of transmission-blocking strategies, with D13D14 being a highly promising vaccine target. In fact, an optimized Pfs230 D13D14 mRNA-lipid nanoparticle (LNP) vaccine generated antibodies that achieved 99.7% transmission-blocking efficiency in standard membrane-feeding assays [18]. A recent report also indicates that the disk-shaped Pfs48/45 engages Pfs230 mainly through a short C-terminal peptide (residues 3107–3122) downstream of D14, rather than solely via the D13–D14 core [195]. Interestingly, parasites that lack this C-terminal fragment, or D13-D14, remain highly infectious in mosquitoes, suggesting that membrane retention mediated by this interaction is not a prerequisite for transmission. The study also structurally characterized three new high-potency epitopes and elucidated an important aspect of potent anti-Pfs230 antibodies: they target membrane-distal epitopes with an approach angle that facilitates C1q binding and C3 deposition, which is essential for complement-dependent transmission-blocking activity. Conversely, non-potent antibodies attach nearer to the parasite membrane and sterically prevent the formation of the complement activation complex [195]. These findings highlight that the antibody approach angle critically influences complement-mediated activity, a subtle regulatory mechanism, and does not merely involve disrupting the protein complex.

The LCCL domain-containing protein family is critical for *Plasmodium* transmission to mosquitoes [196]. These proteins contain several adhesion modules, including the LCCL domain, that mediate complex PPIs [197]. In* P. falciparum*, such proteins are produced only during the gametocytes and form large multi-protein complexes within the PV [198]. Interactions between the LCCL and SR domains stabilize this extracellular net, which is connected to the GPI-anchored protein Pfs48/45 via Pfs230 [199]. Later, when exposed on the gamete surface, the assembly mediates cellular adhesion events, such as microgamete binding to RBCs during exflagellation [191, 200]. Homologous LCCL–lectin domain adhesion-like proteins are not secreted but are accumulated in intracellular organelles known as crystalloids generated in the ookinete stage in *P. berghei* [201]. Functional studies confirm the essential role of these complexes in both species: disrupting the relevant genes in *P. falciparum* impairs sporozoite migration to salivary glands. In contrast, loss of LAP proteins in *P. berghei* disrupts crystalloid formation and sporulation [199, 202]. The assembly of LCCL-based complexes is involved in the production of infectious sporozoites, despite variations in subcellular localization. Even though phenotypes become apparent later, interventions that interfere with adhesive interactions within such protein networks may provide new approaches to transmission blocking.

## Conclusions

Our grasp of *Plasmodium* protein complexes has evolved considerably—from studying isolated interactions to recognizing the integrated molecular machinery that facilitates the parasite’s intricate life-cycle. This review systematically organizes the critical PPIs and multi-subunit complexes involved in both the asexual proliferation and sexual development stages of *Plasmodium* parasites, with the results suggesting that PPI interfaces are a promising resource for identifying stage-specific drug targets, particularly those in the intracellular erythrocytic stages (Table 1). PPI targets are gradually shedding the “undruggable” label and have been drivers of diverse inhibitor development strategies, including phenotypic screening, target-based screening and structure-based design [8]. However, these PPI-induced strategies face certain challenges in clinical applications for malaria prevention and control. Bottlenecks in experimental and computational methods have limited the establishment of a comprehensive PPI map of *Plasmodium*. Studying PPI is complicated by the parasite genome’s high AT content (approx.  80%), which makes heterologous expression difficult, and results obtained by traditional methods often exhibit a very high false-negative rate [203]. Additionally, detecting transient and weak interactions using standard affinity purification–mass spectrometry is challenging, and the membrane protein interaction group has not been well studied due to the lack of a screening platform that mimics physiological conditions. Approximately 30% of *Plasmodium* proteins lack homologous templates, which also hampers inference through homologous mapping. Tools like AlphaFold-Polymers can predict binary complex structures, but they are still not sufficiently accurate to model PPI involving multi-component systems, dynamic assembly or post-translational modifications. Furthermore, the absence of high-resolution complex structures significantly decreases the reliability of calculations and analyses [204–206]. The prediction of existing interaction networks is often influenced by “literature bias,” and many potential functional targets have not been fully explored [207]. Converting PPI-targeted compounds into clinical use is challenging because their physical and chemical properties differ substantially from those of traditional small molecule drugs. The PPI interface is usually large in area, flat in shape and dispersive in binding force. Consequently, effective inhibitors are usually large in terms of molecular weight and lipophilic, which leads to poor oral bioavailability, low solubility and poor stability. These problems hinder the development of long-acting oral drugs suitable for malaria-endemic areas [208, 209].

 It is worth noting that the life-cycle of malaria parasites is characterized by stage heterogeneity; therefore, the ideal drug target must play a central role across its multiple developmental stages, which undoubtedly poses a significant challenge for target verification [210, 211]. In the case of limited resources, prioritizing *Plasmodium* PPI targets requires a multi-dimensional, comprehensive evaluation system. It is not only necessary to analyze the biological necessity, stage coverage and host selectivity of the target through genome-wide metabolic models; at the same time, by analyzing and comparing the metabolic networks of malaria parasites and humans, important parasite-specific targets have been selected [210, 212]. In addition, standardized guidelines developed by consortia such as MalDA are to be applied. Candidate targets must also meet the target product profile criteria and be druggable. Protein complexes that have passed the phenotypic screening test, can cross to interfere with various pathways and exhibit low drug resistance risks should be given priority. In the meantime, jobs can be prescreened for the quality of the target structure and binding potential using computational biology tools, which selectively identify candidate targets with the highest development value [129, 213, 214]. To minimize off-target toxicity, drug design should maximize the exploitation of differences between host and parasite evolution [211]. Strategies involve targeting parasite-specific domains or designing with precision by focusing on subtle amino acid differences in binding pockets within conserved protein complexes like Hsp90 [215]. Of course, entirely parasite-specific interaction interfaces, such as AMA1-RON2, are ideal candidates for minimizing host toxicity [216]. In theory, PPI interfaces are usually subject to stronger evolutionary constraints, and developing drug resistance often requires co-mutation at multiple sites, significantly raising the evolutionary barrier. A more effective approach, however, would be to target multiple key nodes of the PPI network simultaneously—for example, by inhibiting Hsp90 and the PIKK pathway, to further strengthen this barrier and delay the development of drug resistance [122, 217–219].

For the development of antimalarial drugs in PPI, it is necessary to make systematic progress across many aspects. From a chemical design perspective, it is important to move beyond the traditional small-molecule approach and focus on developing macrocyclic compounds, such as cyclic peptides, that can better interact with the broad, flat structural characteristics of the PPI interfaces [208]. From a methodological perspective, combining techniques like Cryo-EM and CRISPR–Cas9-based proximity labeling will be crucial for capturing transient interactions under near-physiological conditions and building high-resolution dynamic PPI maps [14, 220–222]. Simultaneously, combining single-cell RNA sequencing, protein interaction networks and deep learning methods (such as Parker-R2P, scNET, PINNACLE) aids in analyzing molecular-level data, predicting protein abundance at single-cell resolution and generating interaction diagrams that have not been explored before. New technologies, such as MAP-X, are useful tools for target verification [223–227]. To address drug resistance, it is advisable to perform evolutionary experiments early in research and development to anticipate possible resistance pathways. Additionally, promoting multi-target, collaborative intervention strategies is recommended. Finally, by combining computational and experimental methods, specialized tools and databases for analyzing PPIs in *Plasmodium* will be created, with a focus on common challenges such as oral bioavailability, targeting of disordered regions and designing multi-target regulators. Achieving precise and effective intervention on the key interaction networks of parasites is crucial for developing the next generation of antimalarial drugs with novel mechanisms and a high barrier to the emergence of drug-resistant parasites.

**Table 1:** Summary of key protein–protein interaction targets and inhibitory strategies in *Plasmodium* asexual blood- tages

Developmental stage	PPI target/complex	Key components	Biological function	Targeted inhibitors/strategies	Development stage	Reference(s)
Merozoite invasion	Initial attachment complex	MSP-1/6/7 complex	Mediates low-affinity initial contact with the erythrocyte surface	Anti-MSP-1 mAbs	Clinical trials	[29]
	Calcium signaling network	CDPK1, PfPKA	Coordinates microneme secretion and triggers the molecular motor	Pyrazolopyrimidines (BKI-1294, 3-MB-PP1)	In vitro discovery	[68–70]
	PCRCR complex, EBL family, PfRh family	PfRH5-CyRPA-Ripr-PTRAMP-CSS EBA-175, EBA-140, EBA-181, EBL-1, PfRH1, PfRH2a/b,PfRH4	Essential for membrane deformation and triggering calcium flux	Anti-PfRH5	Preclinical animal models	[38, 40, 42, 43, 46–49]
				CyRPA/Ripr mAbs, Plasmepsin X inhibitors (WM4, WM382), Anti-EBA-175 mAb, Anti-PfRH2b mAb	In vitro discovery	
	Moving junction (MJ)	AMA1–RON2 Complex	Facilitates irreversible attachment and committed entry into the host cell	Monoclonal antibodies (4G2, 1F9), Synthetic RON2L peptides, R1, F1	In vitro discovery	[52, 54, 55]
				75B10, 75C8, SBD1	Preclinical animal models	
	Glideosome (motor)	MyoA-MTIP-ELC, GAP45, GAP50	Generates the mechanical force required for parasite internalization	Cytochalasin D, KNX-002, Curine and MMV688271	In vitro discovery	[63–65]
				Brilacidin	Clinical trials	
				Bedaquiline	FDA-approved	
Intraerythrocytic survival	Mitochondrial ETC	bc_1_ complex, PfNDH2, PfDHODH interface	Maintains mitochondrial membrane potential and pyrimidine biosynthesis	CK-2-68, WR249685, 5-fluoroorotic acid	In vitro discovery	[95, 96]
				ELQ-300, SL-2-25, SL-2-64	Preclinical animal models	
				DSM1	Clinical trials	
				Atovaquone	FDA-approved	
	Nuclear import system	PfIMPα-NLS / PfIMPβ1	Mediates nuclear translocation of transcription factors and cargo	GW5074, Auranofin, BAY11-7085	In vitro discovery	[97, 98]
				Ivermectin	FDA-approved	
	Proteostasis machinery	eIF4G1-eIF1, eEF1α/eEF2-ribosome, 70S ribosome, Hsp70-1, TRiC chaperonin, proteasome	Regulates precise protein translation, folding, and degradation	i14G1-12, MAL3, 15-deoxyspergualin, PMB and EGCG, Malonganenones A–C, Lapachol, Bromo-β-lapachone, Geldanamycin, harmine derivatives, purine-based compounds, novobiocin, TPR-domain targeting peptidomimetics, Clemastine	In vitro discovery	[110–114, 116, 117, 121, 125, 132]
				Quinoline-quinazoline dual-substrate inhibitor, J-80, TDI-8304, Celastrol, Artezomibs	Preclinical animal models	
				DDD107498, Cabamiquine	Clinical trials	
				Mefloquine, doxycycline, artemisinin, argatroban, pemetrexed hydrate, atazanavir sulfate, and LM-3632	FDA-approved	
Merozoite egress	Proteolytic cascade	SUB1, Plasmepsin X, SERA6-MSA180	Sequential rupture of the PVM and the host erythrocyte membrane	MRT12113, 3j CWHM-117 E64d, MMV676881, triazine nitriles	In vitro discovery	[135, 137, 139, 140]

*FDA* US Food and Drug Administration,* mAbs* monoclonal antibodies; for other terms, see Abbreviations

## Data Availability

Not applicable.

## Abbreviations

ACDC: AP2-coincident domain
AMA1: Apical membrane antigen 1
APEX2: Ascorbate peroxidase 2
ApiAP2: Apicomplexan AP2
BioID: Biotin identification
CITH: CARP interactor with THY domains
CLAG3: Cytoadherence-linked asexual gene 3
ClpP: Caseinolytic protease P
CoQ: Coenzyme Q/ubiquinone
CPN60: Chaperonin 60
Cryo-EM: Cryo-electron microscopy
DCA: DOZI/CITH/ALBA
DOZI: Development of zygote inhibited
EBL: Erythrocyte-binding-like
ELC: Essential light chain
GAC: Glideosome-associated connector
GAP45/50/40: Glideosome-associated protein 45/50/40
GCα: Guanylyl cyclase α
GDV1: Gametocyte development 1
GEP1: Gametogenesis essential protein 1
GPA: Glycophorin A
HDP: Heme detoxification protein
HP1: Heterochromatin protein 1
IMC: Inner membrane complex
K13: Kelch13
MJ: Moving junction
MSA180: Merozoite surface antigen 180
MSP: Merozoite surface protein
mtETC: Mitochondrial electron transport chain
MTIP: MyoA tail interacting protein
MyoA: Myosin A
NLS: Nuclear localization signal
OB: Osmiophilic body
P-EV: PPLP2-negative egress vesicle
PfDHODH: *Plasmodium falciparum* dihydroorotate dehydrogenase
PfEBA: *Plasmodium falciparum* erythrocyte binding antigen
PfEMP1: *Plasmodium falciparum* erythrocyte membrane protein 1
PfMORC: *Plasmodium falciparum* microrchidia
PfNDH2: *Plasmodium falciparum* NADH dehydrogenase 2
PfRH: *Plasmodium falciparum* reticulocyte-binding homologue
PfSUB2: *Plasmodium falciparum* subtilisin-like protease 2
PPI: Protein–protein interaction
PPM: Parasite plasma membrane
PROTAC: Proteolysis-targeting chimera
PSAC: *Plasmodium* Surface anion channel
PTEX: Plasmodium translocon of exported proteins
PV: Parasitophorous vacuole
PVM: Parasitophorous vacuole membrane
RBC: Red blood cell
RCR: Rolling circle replication
RhopH: Rhoptry H
RON complex: Rhoptry neck protein complex
SERA: Serine repeat antigen
SLF: Signaling linking factor
SUB1: Subtilisin-like protease 1
TRiC/CCT: TCP-1 ring complex/chaperonin containing TCP-1
TRPM7: Transient receptor potential melastatin 7
TurboID: Turbo biotin identification
UGO: Unique GC organizer
uORF: Upstream open reading frame
XA: Xanthurenic acid
